# Background Diet Influences TMAO Concentrations Associated with Red Meat Intake without Influencing Apparent Hepatic TMAO-Related Activity in a Porcine Model

**DOI:** 10.3390/metabo10020057

**Published:** 2020-02-06

**Authors:** Rebekka Thøgersen, Martin Krøyer Rasmussen, Ulrik K. Sundekilde, Sophie A. Goethals, Thomas Van Hecke, Els Vossen, Stefaan De Smet, Hanne Christine Bertram

**Affiliations:** 1 Department of Food Science, Aarhus University, Agro Food Park 48, DK-8200 Aarhus N, Denmark; rebekka.thoegersen@food.au.dk (R.T.); martink.rasmussen@food.au.dk (M.K.R.); uksundekilde@food.au.dk (U.K.S.); 2 Laboratory for Animal Nutrition and Animal Product Quality, Faculty of Bioscience Engineering, Ghent University, Coupure Links 653, 9000 Ghent, Belgium; SophieA.Goethals@UGent.be (S.A.G.); Thomas.VanHecke@UGent.be (T.V.H.); Els.Vossen@UGent.be (E.V.); Stefaan.DeSmet@UGent.be (S.D.S.)

**Keywords:** TMAO, red and processed meat, white meat, background diet, hepatic gene expression

## Abstract

Red meat has been associated with an increased cardiovascular disease (CVD) risk, possibly through gut microbial-derived trimethylamine-N-oxide (TMAO). However, previous reports are conflicting, and influences from the background diet may modulate the impact of meat consumption. This study investigated the effect of red and white meat intake combined with two different background diets on urinary TMAO concentration and its association with the colon microbiome in addition to apparent hepatic TMAO-related activity. For 4 weeks, 32 pigs were fed chicken or red and processed meat combined with a prudent or western background diet. ^1^H NMR-based metabolomics analysis was conducted on urine samples and hepatic Mrna expression of TMAO-related genes determined. Lower urinary TMAO concentrations were observed after intake of red and processed meat when consumed with a prudent compared to a western background diet. In addition, correlation analyses between urinary TMAO concentrations and relative abundance of colon bacterial groups suggested an association between TMAO and specific bacterial taxa. Diet did not affect the hepatic Mrna expression of genes related to TMAO formation. The results suggest that meat-induced TMAO formation is regulated by mechanisms other than alterations at the hepatic gene expression level, possibly involving modulations of the gut microbiota.

## 1. Introduction

Meat constitutes an important part of human diet as a pivotal source of high-quality proteins [1]. In addition, meat consumption contributes with minerals and micronutrients, including highly bioavailable heme-iron, zinc, selenium, and important B vitamins, particularly vitamin B12 [1]. Despite its nutritional value, in recent years, increasing attention to associations between meat consumption and human health has emerged. Prospective studies investigating the association between meat intake and mortality have suggested associations between the consumption of meat and several chronic diseases, including cardiovascular diseases (CVD) and cancer [2,3,4]. However, data are inconsistent, and other studies have found no associations between meat consumption and mortality [5,6] or CVD risk [7]. The conflicting data may be attributed to confounding with other lifestyle factors. This is supported by the fact that several studies conclude that lower disease risk in vegetarians than non-vegetarians cannot be attributed to the absence of meat in the diet but to other factors, such as smoking, socioeconomic status, and health consciousness [8,9,10]. Thus, influences from the background diet can also be expected to modulate the impact of meat consumption.

One of the proposed mechanisms by which meat intake may influence CVD risk involves the formation of trimethylamine-N-oxide (TMAO) during the metabolism of TMAO precursors from meat by the intestinal microbiota that also involves subsequent hepatic oxidation [11]. The first step in TMAO formation is the gut microbial metabolism of TMAO precursors, including carnitine and choline, which results in the formation of trimethylamine (TMA). Subsequently, TMA is oxidized in the liver by flavin monooxygenase enzyme (FMO) family members to form TMAO [11,12]. Koeth et al. (2013) found that circulating TMAO levels were higher for subjects consuming meat than vegetarians and that TMAO accelerated atherosclerosis in mice [11]. An association between meat intake and TMAO has also been indicated in later studies targeted at discovering biomarkers related to red meat intake [13,14,15]. However, other dietary factors, such as fish consumption, also affect plasma TMAO levels [16]. A cross-sectional study found that red meat but not white meat intake was positively associated with plasma TMAO concentrations, and that fish intake was associated with increased TMAO concentrations in both plasma and urine [17]. In addition, studies suggest that gut microbiota composition influences the extent of TMAO production [18,19]. Intriguingly, a recent study where women on a high-meat Paleolithic diet were compared with women on a regular diet concluded that the high-meat Paleolithic diet was not associated with an increase in plasma levels of TMAO [20]. Thus, there is a lack in our understanding of the precise associations between meat intake, background diet, and TMAO production. Using a pig model, the present study aimed, therefore, to investigate the effect of red and white meat intake combined with two different background diets; a high-fiber vegetable background diet and a high-fat low-vegetable background diet, on urinary TMAO concentration and its association with the colon microbiome and on apparent hepatic TMAO-related activity. 

## 2. Results

The present study aimed to investigate the effect of white versus red and processed meat combined with a western high-fat low-vegetable or a prudent high-fiber vegetable background diet on TMAO formation and the correlation with colon microbial relative abundances as well as on hepatic gene expression. At first, multivariate data analysis was conducted on the ^1^H NMR spectroscopic data to explore possible effects of meat source and background diet on the urinary metabolome. Urinary TMAO excretion was quantified, and potential correlations with the available microbial community data at different taxonomic levels were explored. Subsequently, Mrna expression of relevant genes in the liver was determined to study apparent hepatic TMAO-related activity. 

### 2.1. Urinary Metabolome

**Figure 1:**
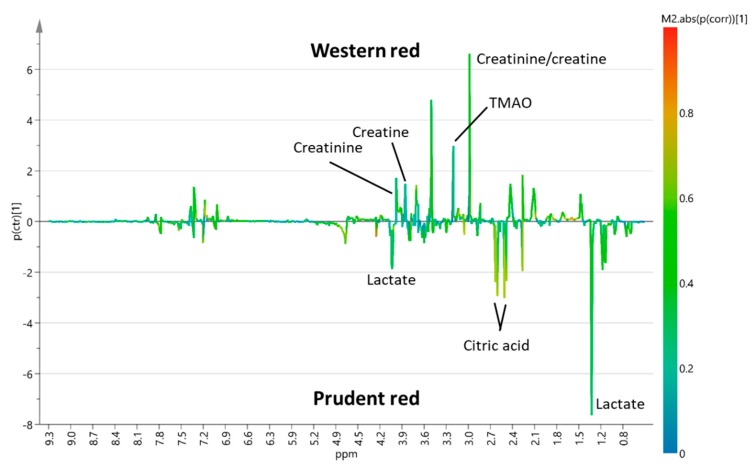
Orthogonal projections to latent structures discriminant analysis (OPLS-DA) S-line plot of pairwise comparison between urinary spectral data obtained from pigs fed red and processed meat combined with a western (n = 7) or prudent background diet (n = 6) (Q^2^ = 0.75).

**Figure 2:**
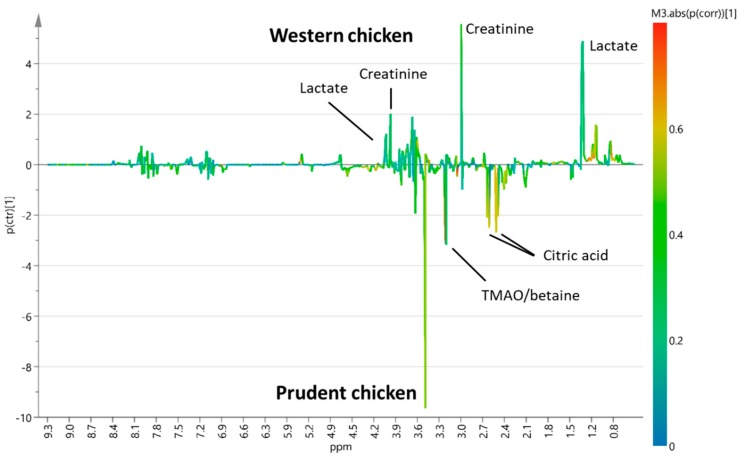
OPLS-DA S-line plot of pairwise comparison of urinary spectral data obtained from pigs fed chicken combined with a western (n = 8) or prudent (n = 6) background diet (Q^2^ = 0.30).

To investigate possible effects of background diet, supervised Orthogonal projections to latent structures discriminant analysis (OPLS-DA) models for pairwise comparison of western versus prudent background diet combined with either red and processed meat or chicken, respectively, were constructed (Supplementary Material S1, Figures S1 and S2). OPLS-DA S-line plots were used to reveal urinary metabolites associated with each of the experimental diets (Figure 1 and Figure 2). 

**Figure 3:**
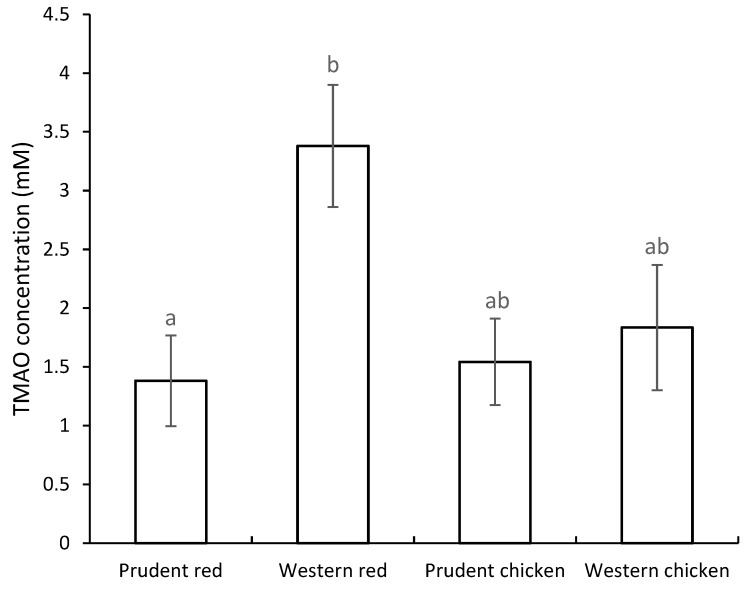
Trimethylamine-N-oxide (TMAO) concentration (mean ± SEM) in urine samples from pigs fed a diet based on chicken or red and processed meat combined with a prudent or western background diet. Prudent red, n = 6; western red, n = 7; prudent chicken, n = 6; western chicken, n = 8. Bars not sharing a common letter are significantly different. Area under the curve of the NMR spectral data did not differ between dietary treatment groups, indicating no effect of diet on the dilution of the urine samples.

OPLS-DA S-line plot comparing red and processed meat intake combined with a western or prudent background diet (Q^2^ = 0.75) indicated increased intensities of peaks assigned to TMAO, creatinine, and creatine after intake of western background diet (Figure 1). In addition, a peak at 3.55 ppm tentatively assigned to glycine showed an increased intensity when red and processed meat was combined with a western background diet. On the contrary, an increased intensity of citrate and lactate was found when red and processed meat was combined with a prudent background diet (Figure 1). OPLS-DA pairwise comparison model of white meat combined with western or prudent background diet showed only modest model predictability (Q^2^ = 0.30) (Figure 2). OPLS-DA indicated increased intensities of lactate and creatinine when chicken intake was combined with a western background diet, whereas chicken intake combined with a prudent background diet showed increased intensities of citric acid, betaine, TMAO, and a peak at 3.55 ppm tentatively assigned to glycine (Figure 2). Two-way ANOVA of quantified TMAO concentrations revealed an effect of background diet on TMAO concentrations (*p* = 0.03). The TMAO concentration in urine from pigs fed red and processed meat combined with a western background diet was significantly higher compared to urine from pigs fed red meat combined with prudent background diet, 3.38 Mm and 1.38 Mm, respectively (Tukey’s honest significant different (HSD) test, *p* = 0.04) (Figure 3). A near-significant difference was found between pigs fed chicken combined with prudent background diet (1.54 Mm) and pigs fed red meat combined with western background diet (3.38 Mm) (Tukey’s HSD test, *p* = 0.06). Metabolite concentrations of the remaining urinary metabolites identified in OPLS-DA S-line plots are given in Supplementary Material S1, Table S1. For betaine, citric acid, creatine, and lactate urinary concentrations did not differ between dietary treatment groups. For creatinine, a significantly higher concentration was found in urine from pigs fed red meat combined with the western background diet (10.14 Mm) compared to the prudent background diet (4.68 Mm) (Tukey’s HSD test, 0.03).

### 2.2. Association between Urinary TMAO and Colon Microbiome

**Figure 4:**
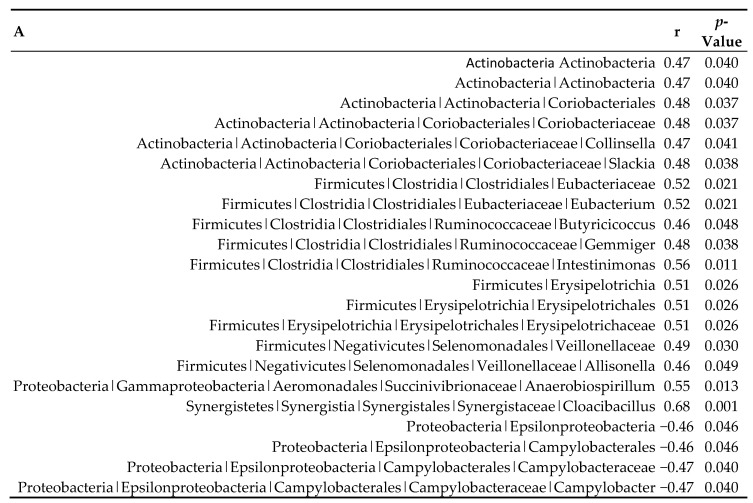

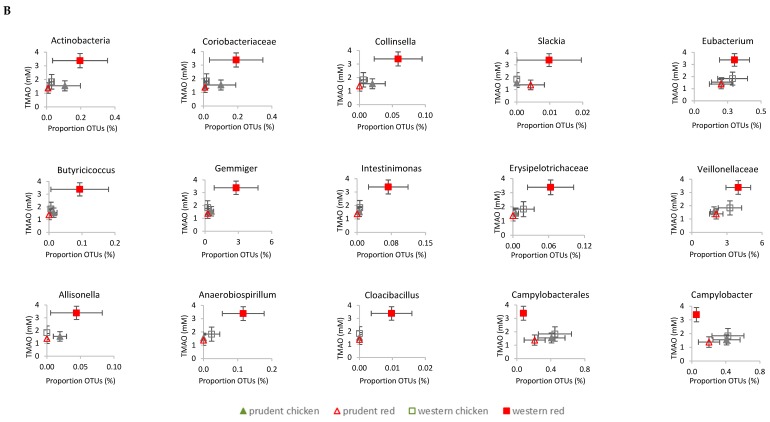
Urinary TMAO concentrations were associated with the colonic microbiome composition of pigs subjected to four dietary treatments. (**A**) Significant Pearson correlations (*p* < 0.05) between TMAO concentrations and the relative abundance of the indicated microbiota in different taxonomic levels. Pearson correlation coefficients (r) and *p*-values are given. Prudent red, n = 5; western red, n = 5; prudent chicken, n = 4; western chicken, n = 5. (**B**) TMAO concentrations and proportion operational taxonomic units (OTUs) grouped by dietary treatment. Data are expressed as mean ± SEM for both TMAO concentrations and the proportion of OTUs.

Correlation analyses indicated an association between multiple microbial taxa and urinary TMAO levels (Figure 4). Various families, such as Coriobacteriaceae, Eubacteriaceae, Erysipelotrichaceae, and Veillonellaceae, and genera, including *Collinsella*, *Slackia*, *Eubacterium*, *Butyricicoccus*, *Gemmiger*, *Intestinimonas*, *Allisonella*, *Anaerobiospirillum*, and *Cloacibacillus* were positively associated with TMAO concentrations (0.47 < r < 0.68), whereas Campylobacteraceae and *Campylobacter* were negatively associated with TMAO levels (r = −0.47). Among the taxa that were significantly associated with TMAO concentrations, the genus *Gemmiger* and family Veillonellaceae had the highest relative abundances. 

### 2.3. Hepatic Gene Expression

Hepatic expression of genes related to TMAO formation, flavin monooxygenase 1 (FMO1), and FMO3 flavin monooxygenase 3, was not affected by diet (Table 1). 

A near-significant effect of background diet was found on mRNA expression of FXR (*p* = 0.06). For CYP7A1 expression, no differences between dietary treatment groups were found. To further evaluate the effect of meat intake and background diet on hepatic gene expression, we also analyzed the mRNA expression of CYP1A2, CYP1B1, CYP2B22, CYP2E1, CYP3A29, and CYP4A24. No effect of diet was found on mRNA expression, except for CYP4A24, where a significant effect of the background diet was found (*p* < 0.01) (Table 1). Therefore, we also evaluated the effect of diet on peroxisome proliferator-activated receptor-alpha (PPAR-alpha), the nuclear receptor mediating induction of CYP4A enzymes [21]. A two-fold increased CYP4A24 gene expression in pigs fed red and processed meat combined with western background diet compared to pigs fed chicken combined with prudent background diet (*p* = 0.02) was found in addition to a near-significant increase in pigs fed red and processed meat combined with western background diet compared to prudent background (*p* = 0.06). Two-way ANOVA revealed an effect of background diet on PPAR-alpha mRNA expression (*p* = 0.02). However, Tukey’s HSD test did not reveal significant differences between the dietary treatment groups.

## 3. Discussion

TMAO has attracted attention as a metabolite associated with CVD risk [12]. Red meat contains high amounts of carnitine [22,23], which is a TMAO precursor, and increased circulating TMAO levels and urinary excretion have been observed after red meat consumption [13,15,24]. Thus, TMAO formation might be one mechanism contributing to the proposed association between red and processed meat intake and increased CVD risk [2]. The present study revealed that urinary TMAO concentration following red and processed meat intake is affected by the type of background diet consumed in combination with the red and processed meat-based diet. Thus, a lower urinary TMAO excretion was found in pigs fed red and processed meat combined with a prudent high-fiber vegetable background diet compared to a western high-fat low-vegetable background diet consumed in combination with the same meat-based diet. This outcome could be related to the interaction between diet, host, and microbiome, such as the inhibition in microbial TMA production, shifts in the microbial community, or differential hepatic TMAO-related gene expression. 

### 3.1. Background Diet Affects Urinary TMAO Excretion Following Red Meat Consumption

The present findings showing that background diet influences TMAO formation are striking, as factors decisive for meat-induced TMAO formation are still not well understood. A high meat intake as part of a Paleolithic diet was found not to increase TMAO formation in healthy women compared to the consumption of a regular diet containing lower amounts of red meat and eggs [20]. Wang et al. (2019) recently investigated the effect of red meat, white meat, or non-meat protein intake in the context of a diet high or low in saturated fats on TMAO metabolism in humans. Red meat consumption increased systemic TMAO levels, but high versus low levels of saturated fat in the context of a red meat diet did not affect plasma TMAO or TMAO precursors [24]. Li et al. (2017) investigated the effect of soluble dietary fiber on TMA metabolism in mice fed red meat and found that soluble dietary fibers reduced TMA and TMAO metabolism and modified the gut microbiota in mice fed with red meat [25]. Background diets in the present study were characterized by a complex composition and varied in several parameters. Therefore, the effect on TMAO formation is likely a result of a combination of various factors. Previously, a diet resembling a western diet high in saturated fats and sugar was shown to increase plasma TMAO concentrations in mice compared to the consumption of a standard diet [26]. Likewise, high-fat diets were found to increase postprandial plasma TMAO concentrations in both mice and human [27,28]. It was suggested that a high-fat diet could possibly increase TMAO concentrations through alterations in gut microbial composition and function [26,27,28]. The higher fat content in the western background diet compared to the prudent background diet might have contributed to increased TMAO formation through alterations of the gut microbiota. However, other studies showed no effect of increased amounts of saturated fat in the diet on plasma or serum TMAO concentration [24,29]. The prudent background diet contained higher amounts of fruits and vegetables compared to the western background diet. In fact, the effect of naturally occurring plant compounds on TMAO or trimethylamine (TMA) formation has been investigated in animal models and in vitro. Phytochemicals were found to reduce TMAO formation through modulation of the gut microbiota in mouse models [30,31], and polyphenol-rich plant-based foods were found to reduce the biotransformation of choline and carnitine into TMA in a colonic in vitro model [32]. However, in the latter study, carbohydrates, predominantly in the form of free sugars, possibly caused the main effect [32]. Wu et al. (2015) found that the addition of a food-derived antimicrobial compound, such as allicin found in garlic, can prevent modulation of the gut microbiota induced by a carnitine-enriched diet [31]. Additionally, the structural choline analog, 3,3-dimethyl-1-butanol (DMB), naturally found in foods, such as cold-pressed extra virgin oil, grape seed oil, and some balsamic vinegars, was able to inhibit microbial TMA lyase activity, thereby preventing TMA production [33]. Thus, the high content of fruits and vegetables in the prudent background diet might contribute to the attenuation of TMAO formation. Likewise, the higher fiber content in the prudent background diet may lead to a reduced TMAO formation [25]. Only a few studies have investigated the effect of red versus white meat consumption on urinary TMAO excretion, and urinary TMAO levels do not necessarily correspond to plasma TMAO levels as shown, for example, by Bielinska et al. (2018) [34] and Jakobsen et al. (2017) [15]. A human intervention study comparing diets rich in fish, chicken, red meat and processed meat, respectively, did not report significant differences in urinary TMAO levels between chicken, red meat and processed meat diets [35]. However, a study using a rat model found elevated urinary TMAO levels after consumption of red meat compared to white meat [15]. Likewise, a randomized-controlled dietary intervention found that chronic red meat intake, but not white meat intake, increased urinary TMAO excretion as well as plasma TMAO in human [24]. Findings in the present study disclose that the dissimilarities seen in these former studies likely can be attributed to variations in background diet as we only found differences in TMAO excretion between white and red meat when the background diet was a western-like diet. 

### 3.2. Urinary TMAO Concentrations Correlated with Colonic Bacterial Taxa

The activity of the gut microbiota plays a substantial role in endogenous TMAO formation, as it was previously shown in germ-free mice that the presence of gut microbiota is crucial to form TMAO from oral choline or carnitine administration [11,12]. Nevertheless, the organisms responsible for the metabolization of carnitine and choline to TMA remain largely unknown. Several studies have investigated associations between urinary or plasma TMAO levels and the gut microbiome composition by comparing low-versus high-TMAO producers or low-versus high-meat diets [11,18,30,33,36,37]. The positive correlation between TMAO and several members of Firmicutes in the present study, and not Bacteroidetes species, the two most predominant phyla in the colon of pigs, is in line with previous studies demonstrating a greater enrichment of Firmicutes relative to Bacteroidetes in high-TMAO producers [18,36]. The present study indicated associations between multiple specific taxa and TMAO. Some of these have also been reported in previous studies and include Erysipelotrichaceae [33], Veillonellaceae [37], Coriobacteriaceae [33], and *Colinsella* [38]. Interestingly, *Colinsella* was also found to occur at higher abundances in patients with symptomatic atherosclerosis [39]. However, healthy control patients in the latter study showed enrichment in *Eubacterium*, a genus that was positively correlated with TMAO levels in the present study. The genus *Ruminococcus* or uncultured Ruminococcaceae have been frequently described to correlate with TMA and/or TMAO levels [30,33,36] and with aortic lesion size in mice [33]. Although no association with the *Ruminococcus* genus was detected in the present study, several correlations with other genera of the Ruminococcaceae family (*Butyricicoccus*, *Gemminer*, and *Intestinmonas*) were revealed. Next to the assessment of correlations, other studies focused on the key genes encoding for enzymes involved in TMA production to gain more insight into the TMA-forming potential of microbial communities [40,41]. This gene-targeted approach resulted in the characterization of TMA-producing bacterial taxa, such as species from the Coriobacteriaceae family [40], of which some were also found in the present study. On the other hand, many positive or negative correlations, such as the negative association between TMAO with Bifidobacteriaceae found in previous studies [30,37], were not observed in the present study. The observed correlations in this exploratory approach, investigating potential relationships between TMAO generation and the microbial community, require a careful interpretation because of the borderline significance of the correlations and the absence of correction for multiple testing. A larger sample size in future studies would increase the power and significance of the results. Moreover, correlations as such do not imply causation, and follow-up studies are needed to investigate causality. For example, the negative correlation between *Campylobacter* and TMAO in this study is probably due to the association between *Campylobacter* and chicken meat intake [42] and is likely not directly related to TMAO formation.

### 3.3. Effect of Diet on Hepatic TMAO-Related Gene Expression

Flavin monooxygenases play an important role in hepatic TMAO formation. The flavin monooxygenases FMO1 and in particular FMO3 are able to oxidize the gut microbiota-derived metabolite, TMA, into TMAO in the liver [43]. To investigate the underlying mechanisms for endogenous TMAO formation following the ingestion of the four experimental diets, we, therefore, investigated apparent hepatic TMAO-related gene expression. Despite the effect of background diet on urinary TMAO excretion, no effect of diet was found on mRNA expression of FMO3 or FMO1. In agreement with our findings, using a mouse model, Bennett et al. (2013) [43] found that an increased plasma TMAO concentration following a choline-enriched diet was not associated with a concurrent increase in hepatic FMO3 mRNA expression or increased FMO activity. Hence, factors other than FMO expression, including post-transcriptional regulation, may also affect the amount of TMAO formed endogenously [43]. Alternatively, the duration of the intervention might have affected the observed mRNA expression, since an up- or downregulation could have occurred earlier during the intervention until sufficient protein expression levels were reached. FMO3 expression has been found to be regulated by the nuclear receptor, farnesoid X receptor (FXR), which can be induced by bile acids [43]. TMAO has been suspected to increase CVD risk by affecting bile acid metabolism and, thereby, cholesterol elimination [11]. Hence, TMAO supplementation reduced hepatic expression of bile acid transporters and bile acid synthetic enzymes, including CYP7A1, as well as reduced cholesterol absorption in mice [11]. Therefore, we also investigated the effect of experimental diets on hepatic FXR and CYP7A1 mRNA expression. No effect of diet was found on CYP7A1 mRNA expression, whereas a near-significant effect (*p* = 0.06) of meat source on FXR mRNA expression was observed, indicating a tendency of increased FXR expression following red meat intake. Although not significant, this could indicate that the TMAO formation was, to some extent, affected by the expression of FXR. However, other factors likely affected TMAO formation to a greater extent. In line with the above-mentioned correlations to colonic microbial bacteria, this could likely involve gut microbial mechanisms. As the liver is the major expression site for the cytochrome p450 and FXR is involved in the regulation of several CYP enzymes [44,45], we also as an explorative approach, investigated the effect of diet on the expression of several CYP enzymes, including target genes of other major transcription factors controlling the CYP transcription. Previous studies found that high-fat diet consumption affected the expression of various CYPs [46,47,48,49]. Among these, increased mRNA expression of CYP4A enzymes has been found following high-fat diet consumption [46,48]. The induction of CYP4A enzymes is mediated by the nuclear receptor PPAR-alpha [21]. In line with this, Patsouris et al., 2006 also found that the high-fat diet induced an increase in mRNA expression of CYP4A in mice was PPAR-alpha dependent [48]. In accordance with these studies, we also found an effect of background diet on CYP4A24 mRNA expression. Thus, the high-fat western background diet tended to increase CYP4A24 mRNA expression compared to the prudent background diet. In addition, background diet also affected PPAR-alpha expression towards a higher mRNA expression after consumption of the western background diet, although Tukey’s HSD test revealed no significant differences between the groups. This finding indicates that the increase in mRNA expression of CYP4A24 in the present study was mediated by PPAR-alpha. For the remaining investigated CYP enzymes, we observed no effect of the diets on the expression of these genes. Therefore, it is suggested that general detoxification and phase I metabolism is not affected by meat type or diet background. 

### 3.4. Effect of Background Diet on Other Metabolites

In addition to TMAO, background diet was also shown to affect the urinary excretion of other metabolites when combined with red meat, as indicated by the OPLS-DA S-line plot (Figure 1), although for many metabolites, significance was not reached for the quantified concentrations. Thus, creatinine was also increased upon a western background diet combined with red meat intake, and creatine likewise tended to be increased (P_BD_ = 0.1, Table S1). Creatine is present in animal muscles, and the main dietary source is meat, including red meat, fish, and poultry [50]. Several studies have reported an increase in creatine levels in urine after meat intake [13,51,52], and Pallister et al. proposed circulating levels of creatine as a possible marker of habitual intake of red meat and poultry, as observed in the UK Twin cohort study (n = 3559 subjects) [53]. In relation to the use of creatine as a biomarker of meat intake, it is intriguing that the present study indicates that background diet could have an impact on the effect of red meat on creatine excretion, as it reveals that creatine cannot be used quantitatively without additional information on the diet composition. In the present study, the OPLS-DA S-line plot also revealed that urinary excretion of citric acid was increased upon a prudent diet, and two-way ANOVA of the quantified data indicated a near-significant effect of background diet (P_BD_ = 0.07, Table S1). A former biomarker study has identified citrate as a biomarker of intake of a Mediterranean diet [54], and Xu et al. (2010) found that the most influential metabolites characterizing vegetarians relative to the omnivorous included citrate [55]. Consequently, consistent with former studies, the present study suggests that citrate may be a urinary biomarker of a diet high in vegetables. According to the OPLS-DA S-line plot, a prudent diet was also found to be associated with increased urinary excretion of lactate when combined with red meat intake, whereas for white meat intake, a western background diet appeared to be associated with urinary lactate excretion. However, when quantified, lactate concentrations did not differ between the dietary treatment groups, likely due to large inter-individual variations. The origin of this effect remains unknown, but it may reflect an effect directly on glucose metabolism [56] or glucogenic amino acid metabolism [57]. In conclusion, the consumption of a prudent background diet reduced red meat-related formation of TMAO without significantly altering hepatic mRNA expression related to TMAO formation. Overall, the data suggest that TMAO generation related to red meat ingestion, and the effect of background diet is merely regulated by gut microbial activity than modulation of hepatic capacity at the gene level. Future studies in humans would be relevant to gain further knowledge on interactions between background diet and TMAO generation.

## 4. Materials and Methods

### 4.1. Experimental Design and Diets

The study was conducted using a 2 × 2 factorial design and consisted of the following four diets: (1) red and processed meat combined with a prudent background diet, (2) red and processed meat combined with a western background diet, (3) chicken meat combined with a prudent background diet, (4) chicken meat combined with a western background diet. Diet macronutrient composition is provided in Supplementary Material S1, Table S2, and a detailed description of the composition and preparation of the diets have previously been published [58]. The diets were formulated to mimic the complexity of human diets by consisting of a large variety of food items and different preparations methods. The four diets provided equal intake of metabolizable energy and meat intake per day. 

For the groups receiving red and processed meat, the meat fraction was composed of 62% fresh meat (mainly pork and beef) and 38% processed meat (mainly cooked ham, filet de sax, salami, and smoked bacon). For the groups receiving chicken, the meat fraction was composed of chicken thighs and chicken breasts in addition to chicken skin in order to obtain isocaloric diets. The meat content was 291 g meat/day corresponding to a daily intake of “meat, fish, eggs, and meat alternatives” for the 97.5 percentile of the Belgian population older than 15 years, as reported by the Belgian Food Consumption Survey 2004 [59].

The western background diet was characterized by high amounts of refined grains, desserts, and sweets, whereas the prudent background diet contained high amounts of fruits, vegetables, whole grains, and dairy products. The western background diet was formulated based on the average food intake of the Belgian population [59], and the prudent background diet was based on the dietary guidelines of the Flemish active food guide pyramid [60]. The Nubel databank was used to design and estimate the nutritional content of the diets [61]. All food items were mixed and prepared to obtain homogenous feed mixtures [58]. 

### 4.2. Animals and Sampling

Thirty-two castrated male piglets (Topigs × Piétrain) at three weeks of age, originating from eight litters, were purchased from a local commercial farm. The pigs were allocated to eight pens and fed a commercial prestarter diet ad libitum for 1 week. Then, during the following week, the experimental diets were gradually introduced, and the piglets were fed the experimental diets during the following four weeks. The four piglets of each litter were divided between the four experimental diets, and each of the four experimental diets were repeated in two pens (n = 8 per diet). Body weight was measured twice a week, and water was available ad libitum at all times. Experimental diets were fresh meals served ad libitum for 30 min at room temperature three times a day (8, 12, 18 h). Individual feed intake per meal was recorded using an individual feeding system. After the four weeks of intervention, pigs were euthanized, and sampling was conducted. Due to practical reasons, euthanasia and sampling was spread over four consecutive days, where two piglets from each dietary group were slaughtered each day, always starting with the heaviest piglets. Piglets were euthanized by intramuscular injection of xylazine (20 mg/mL, 0.1 mL/kg body weight) and intraperitoneal injection of pentobarbital (60 mg/mL, 0.8 mL/kg body weight) two hours after receiving the last meal. Urine was taken with a syringe from the bladder (after euthanasia), filtered over glass wool, divided in aliquots, and stored at −80 °C. The liver (lobus hepatis dexter medialis) was removed, gently rinsed with 0.9% NaCl solution, manually chopped and homogenized before storage at −80 °C. The large intestinal content was collected, gently homogenized, and stored at −80 °C to investigate the correlation between the colon microbiome composition and TMAO concentrations. The effect of dietary treatment on the colon microbial community is explored in another study [62]. 

The study was approved by the Ghent University Ethical Committee at the Faculty of Veterinary Medicine (EC 2016/26). 

### 4.3. H Nuclear Magnetic Resonance (NMR) Spectroscopy

For ^1^H NMR spectroscopy of urine samples, the following number of samples were available: western red, *n* = 7; prudent red, *n* = 6; western chicken, *n* = 8; prudent chicken, *n* = 6. Prior to analysis, urine samples were thawed and prepared by brief vortex mixing, and 600 µL sample was centrifuged at 4 °C (10,000 *g* for 5 min). Subsequently, 500 µL supernatant, 100 µL 0.75 M phosphate buffer in D_2_O and 25 µL D_2_O with 0.05 wt. % 3-(trimethylsilyl)propionic-2,2,3,3-d_4_ acid (TSP; Sigma-Aldrich, St. Louis, MO, USA) were transferred to a 5-mm NMR tube. ^1^H NMR spectroscopy was conducted using a Bruker Avance III 600 MHz spectrometer equipped with a ^1^H TXI probe and operating at a frequency of 600.13 MHz (Bruker BioSpin, Rheinstetten, Germany). A one-dimensional (1D) nuclear Overhauser enhancement spectroscopy (NOESY)-presat pulse sequence (noesypr1d) with pre-saturation to suppress the water resonance was applied. A target temperature of 298 K and the following acquisition parameters were used: number of scans (NS) = 64, spectral width (SW) = 7288.63 Hz, data points (TD) = 32,768, and relaxation delay (D1) = 5 s. Following acquisition, the free induction delays (FIDs) were multiplied by a line-broadening function of 0.3 Hz and Fourier transformed. The obtained spectra were baseline- and phase-corrected in Topspin 3.0 (Bruker BioSpin), and data were imported to MATLAB R2018B (Mathworks Inc., Natick, MA, USA). The interval correlation-shifting algorithm, icoshift [63], was used to reference spectra to the TSP signal (0.0 ppm) and correct for chemical shifting. Signal regions for water (4.76–4.84 ppm), urea (5.6–6.0 ppm), as well as regions below 0.6 ppm and above 9.4 ppm, were removed, and spectra were normalized to the total area and subsequently divided into 0.01 ppm regions. For multivariate data analysis, data were imported to SIMCA 15.0 (Sartorius Stedim Data Analytics AB, Umeå, Sweden), and principal component analysis (PCA) was conducted following pareto scaling. Orthogonal projections to latent structures discriminant analysis (OPLS-DA) models using cross-validation with seven segments were constructed. Metabolites were assigned, and TMAO quantified using Chenomx NMR Suite 8.3 (Chenomx Inc., Edmonton, AB, Canada) using TSP as the internal standard. ^1^H NMR spectroscopy spike-in experiment using TMAO (Sigma-Aldrich) was used to verify the identification of TMAO in urine samples. 

### 4.4. Microbial Community Analysis

Assessment of the colon microbial community using 16S rRNA gene amplicon sequencing was carried out as described by Vossen et al. 2020 [62] for the following number of samples: western red, *n* = 6; prudent red, *n* = 7; western chicken, *n* = 5; prudent chicken, *n* = 6. Briefly, DNA extraction was carried out, followed by amplicon sequencing on an Illumina MiSeq platform. Processing the raw Illumina data resulted in operational taxonomic units (OTU) tables (Supplementary Material S2) that were used to unravel potential correlation with urinary TMAO concentrations in the present paper. 

### 4.5. Real-Time Polymerase Chain Reaction (RT PCR)

RNA extraction was conducted using TriReagent according to the manufacturer’s instructions (Sigma–Aldrich) from 10–20 mg liver samples, while cDNA was synthesized using the iScript cDNA synthesis kit according to the manufacturer (Bio-rad). Taqman-probe-based real-time PCR was conducted as previously described [64] using the StepOnePlus Equipment (Applied Biosystems). The following conditions were used: 2 min at 50 °C, 10 min at 95 °C, followed by 40 cycles at 95 °C for 15 s and 1 min at 60 °C. Specific primer and probes were designed using pig-specific sequences obtained from Ensemble (Sscrofa 11.1), according to Knudsen et al. 2018. [46] The primer and probe sequences of CYP1A2, CYP2B22, CYP2E1, and CYP3A29, and β-actin have been given earlier [65], and the remaining sequences are given in Supplementary Material S1, Table S3. 

Relative mRNA expression was normalized against the geometric mean of the mRNA expression of β-actin and RPLP1. No difference between groups in mRNA expression of the housekeeping genes, nor the geometric mean of the two, was found. Results are given as mRNA expression relative to the average mRNA expression of the prudent chicken diet group, which was set to 1. 

For gene expression analysis, one sample belonging to the group fed red and processed meat combined with a western background diet was identified as an outlier based on PCA scores plot of gene expression data. Hotelling’s T^2^ plot revealed a T^2^ range value for the specified sample larger than the 99% confidence interval limit. Based on this, the sample was excluded from further analyses of hepatic gene expression. 

### 4.6. Statistical Analysis

Mean values were compared using two-way ANOVA to assess the effect of meat source, background diet, and the interaction between meat source and background diet. Two-way ANOVA was followed by Tukey’s honest significant different (HSD) test, and *p*-values < 0.05 were considered significant. Data are given as mean ± SEM. Two-way ANOVA and Tukey’s honest significant different (HSD) test were conducted in MATLAB R2018b (Mathworks Inc., Natick, MA, USA). Associations between urinary TMAO concentrations and relative abundances of individual taxa of the colon microbiome were assessed by calculating Pearson correlations using SAS Enterprise Guide 7. Combination of the available data on urinary TMAO concentrations (N = 27) and microbiome analysis (N = 24) resulted in 19 related observations (prudent red, *n* = 5; western red, *n* = 5; prudent chicken, *n* = 4; western chicken, *n* = 5). *p*-values < 0.05 were considered significant, and all data are given as mean ± SEM.

## Figures and Tables

**Table 1:** Relative mRNA expression (mean ± SEM) in liver tissue obtained from pigs fed a diet with red and processed meat or chicken combined with a prudent or western background diet. N = 8 for each dietary treatment group, except western red where n = 7. Mean values are relative to prudent chicken set to 1. *p*-values indicate the effect of meat source (P_M_), background diet (P_BD_), and the interaction between meat source and background diet (P_M_ × _BD_). For CYP4A24, values not sharing a common superscript letter (a,b) are significantly different.

	Prudent Chicken	Western Chicken	Prudent Red	Western Red	P_M_	P_BD_	P_M_ × _BD_
FMO1	1.00 ± 0.20	1.12 ± 0.12	0.98 ± 0.12	1.32 ± 0.37	0.69	0.29	0.62
FMO3	1.00 ± 0.16	0.89 ± 0.10	1.13 ± 0.25	1.44 ± 0.33	0.13	0.65	0.34
FXR	1.00 ± 0.14	1.00 ± 0.12	1.11 ± 0.15	1.50 ± 0.19	0.06	0.20	0.21
CYP1A2	1.00 ± 0.16	1.58 ± 0.42	1.05 ± 0.18	1.36 ± 0.41	0.79	0.16	0.66
CYP1B1	1.00 ± 0.20	1.41 ± 0.36	1.46 ± 0.41	1.98 ± 0.85	0.30	0.35	0.91
CYP2B22	1.00 ± 0.35	1.37 ± 0.50	1.08 ± 0.26	1.17 ± 0.41	0.87	0.56	0.72
CYP2E1	1.00 ± 0.11	0.88 ± 0.09	0.91 ± 0.10	0.92 ± 0.07	0.80	0.55	0.49
CYP3A29	1.00 ± 0.17	0.76 ± 0.09	0.74 ± 0.16	0.76 ± 0.15	0.38	0.46	0.38
CYP4A24	1.00 ± 0.09 ^a^	1.62 ± 0.22 ^ab^	1.15 ± 0.19 ^ab^	2.02 ± 0.36 ^b^	0.23	<0.01	0.59
CYP7A1	1.00 ± 0.24	1.62 ± 0.86	1.55 ± 0.82	2.73 ± 1.83	0.43	0.39	0.79
PPAR-alpha	1.00 ± 0.09	1.19 ± 0.15	0.96 ± 0.09	1.42 ± 0.20	0.50	0.02	0.34

## Supplementary Materials

The following are available online at https://www.mdpi.com/2218-1989/10/2/57/s1, Supplementary Material S1: Figure S1: OPLS-DA scores plot of urine samples from pigs fed red and processed meat combined with a western (yellow) or prudent (blue) background diet, Q^2^ = 0.75, Figure S2: OPLS-DA scores plot of urine samples from pigs fed chicken combined with a western (red) or prudent (green) background diet, Q^2^ = 0.30, Table S1: Concentrations of metabolites in urine, Table S2: Formulated and analyzed nutritional composition of the four diets, Table S3: Primer and TaqMan probe sequences used for real-time PCR. Supplementary Material S2: OTU table of the colonic microbiome.
