# Supplementary material for: Background Diet Influences TMAO Concentrations Associated with Red Meat Intake without Influencing Apparent Hepatic TMAO-Related Activity in a Porcine Model
Source: Metabolites. 2020 Feb 6;10(2):57. doi: 10.3390/metabo10020057 (PMC7074160; doi:10.3390/metabo10020057)
Supplement: Supplementary file 1 [file metabolites-10-00057-s001.zip › Revised_Supplementary Material 1_TMAO_Metabolites.docx]

**Supporting information 1**


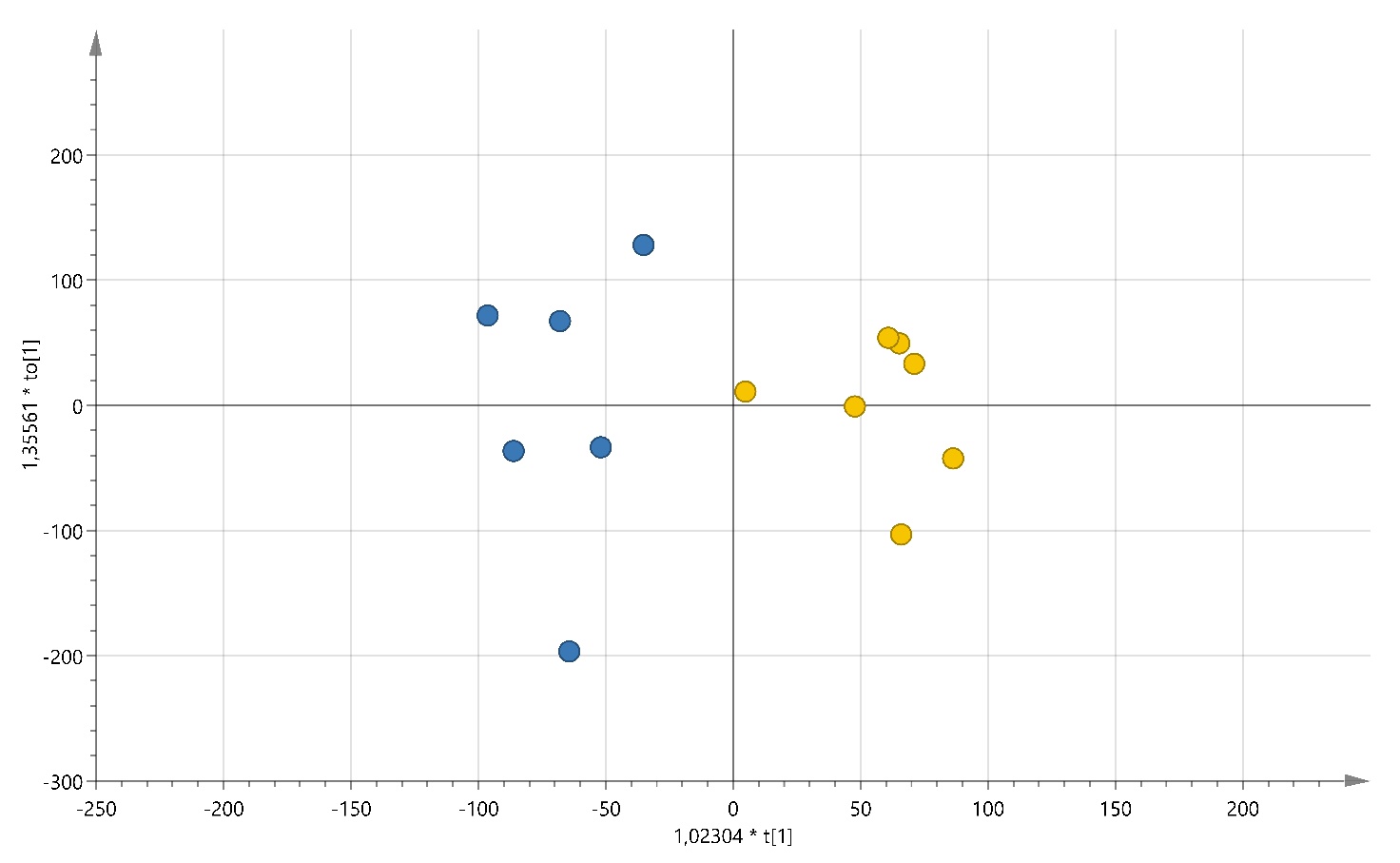


Figure S1. OPLS-DA scores plot of urine samples from pigs fed red and processed meat combined with a western (yellow) or prudent (blue) background diet, Q^2^ = 0.75.


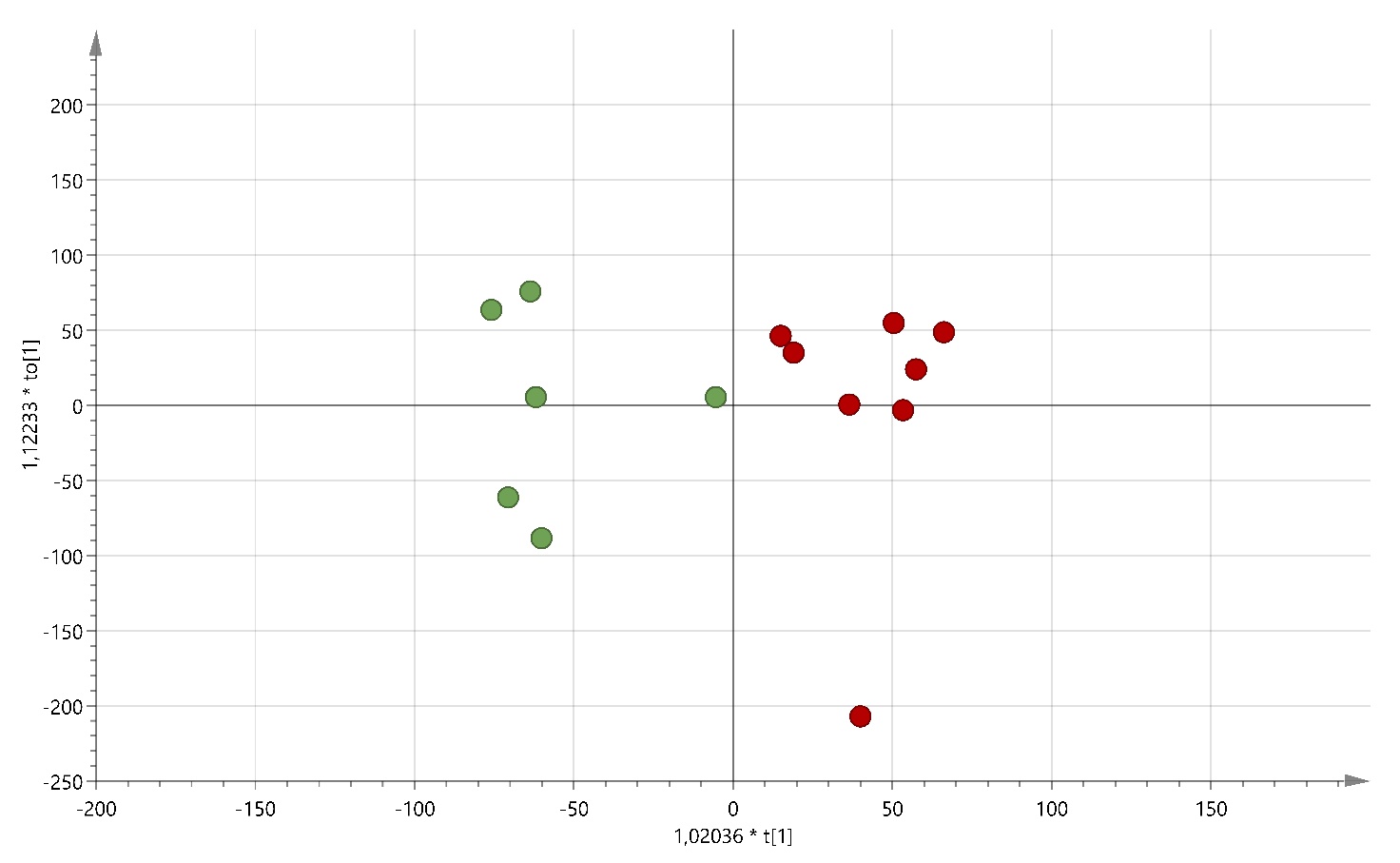


Figure S2. OPLS-DA scores plot of urine samples from pigs fed chicken combined with a western (red) or prudent (green) background diet, Q^2^ = 0.30.

Table S1. Concentration (mM) of metabolites identified in urine samples (mean ± SEM). Values not sharing a common letter within each row are significantly different. P-values indicate the effect of meat source (P_M_), background diet (P_BD_) and the interaction between meat source and background diet (P_M*BD_). Area under the curve of the NMR spectral data did not differ between dietary treatment groups, indicating no effect of diet on the dilution of the urine samples.

|  | Prudent chicken | Western chicken | Prudent  red | Western  red | P_M_ | P_BD_ | P_M*_  _BD_ |
| --- | --- | --- | --- | --- | --- | --- | --- |
| *Betaine* | 0.91 ± 0.57 | 0.18 ± 0.04 | 0.40 ± 0.13 | 0.77 ± 0.37 | 0.91 | 0.58 | 0.10 |
| *Citric acid* | 2.87 ± 1.19 | 0.94 ± 0.42 | 1.35 ± 0.49 | 0.85 ± 0.22 | 0.22 | 0.07 | 0.27 |
| *Creatine* | 0.16 ± 0.06 | 0.78 ± 0.69 | 0.39 ± 0.23 | 2.69 ± 1.38 | 0.22 | 0.10 | 0.33 |
| *Creatinine* | 6.60 ± 0.58^ab^ | 6.92 ± 1.27^ab^ | 4.68 ± 1.40^a^ | 10.14 ± 1.40^b^ | 0.61 | 0.03 | 0.05 |
| *Lactate* | 1.11 ± 0.43 | 9.23 ± 8.30 | 14.40 ± 11.53 | 2.11 ± 1.34 | 0.67 | 0.78 | 0.17 |

*Table S2. Formulated and analyzed nutritional composition of the four diets.*

|  |  | **Formulated** | | |  |  | **Analyzed** | | |  |
| --- | --- | --- | --- | --- | --- | --- | --- | --- | --- | --- |
|  | **Prudent** | |  | **Western** | | **Prudent** | |  | **Western** | |
|  | **Chicken** | **R&P^a^** |  | **Chicken** | **R&P^a^** | **Chicken** | **R&P^a^** |  | **Chicken** | **R&P^a^** |
| **Energy^b^ (Kcal/Kg)** | 1089 | 1089 |  | 1482 | 1482 | 1263 | 1265 |  | 1743 | 1726 |
| **Proportion of energy from proteins (%)** | 22.6 | 23.8 |  | 18.1 | 19.3 | 20.7 | 20.4 |  | 17.7 | 16.4 |
| **Proportion of energy from fat (%)** | 29.2 | 28.8 |  | 40.2 | 39.6 | 22.5 | 24.9 |  | 32.5 | 35.6 |
| **Crude protein (g/100 g)** | 5.99 | 6.40 |  | 6.57 | 7.15 | 6.44 | 6.35 |  | 7.61 | 6.97 |
| **Crude fat (g/100 g)** | 3.45 | 3.46 |  | 6.53 | 6.54 | 3.13 | 3.47 |  | 6.24 | 6.76 |
| **Dry matter (g/100 g)** | 25.5 | 24.7 |  | 33.3 | 32.2 | 26.0 | 26.1 |  | 30.9 | 31.2 |

^a^ R&P = red & processed meat

^b^ Energy of the formulated diets corresponds with metabolizable energy and was estimated by the use of Atwater factors, whereas the analyzed energy corresponds with gross energy as determined by the amount of heat that was liberated in a bomb calorimeter.

Table S3. Primer and TaqMan probe sequences used for real time PCR.

| Name | Sequence 5’-3’ |
| --- | --- |
| FMO1 Forward primer | GGCTTAATACCCGAAGACAGGAT |
| FMO1 Reverse primer | TACGGCCTGGGAGCTCATC |
| FMO1 Probe | CAACTAAGAGAGCCTGTGCT |
| FMO3 Forward primer | GATGAGCTCCCAGCTTGCA |
| FMO3 Reverse primer | ACTCCCTCACATTTGGCTTAATG |
| FMO3 Probe | TCTACGTGGCACCGTGT |
| FXR Forward primer | GCTCTGCTTACAGCAATTGTTATCC |
| FXR Reverse primer | TCTACTGCCTCTCGGTCCTTTATG |
| FXR Probe | CTCTCCAGACAGACAAT |
| CYP7A1 Forward primer | CAGCGACCTTCCGGAATCTA |
| CYP7A1 Reverse primer | CGTGGCCTAGCCCTGAAGT |
| CYP7A1 Probe | ACGACAGCTCAGCTAA |
| CYP4A24 Forward primer | TCCCTGATGGACGCTCCTTA |
| CYP4A24 Reverse primer | GGTTGTGGTGAAGGCCGTAA |
| CYP4A24 Probe | CTGCAGGAATCACCCTCTCGCTCTCC |
| RPLP1 Forward primer | CGGAGCTCGCCTGCAT |
| RPLP1 Reverse primer | TTATCCTCCGTGACCGTAACCT |
| RPLP1 Probe | ACTCTGCCCTCATTCTGCACGACGA |
| PPAR-alpha Forward primer | CGGGAAAGGCCAGCAAT |
| PPAR-alpha Reverse primer | GGCCACGAGCGTCTTCTC |
| PPAR-alpha Probe | ACCCGCCTTTCGTCATACACGACATG |
